# Benthic responses to an Antarctic regime shift: food particle size and recruitment biology

**DOI:** 10.1002/eap.1823

**Published:** 2019-01-02

**Authors:** Paul K. Dayton, Shannon C. Jarrell, Stacy Kim, P. Ed Parnell, Simon F. Thrush, Kamille Hammerstrom, James J. Leichter

**Affiliations:** ^1^ Scripps Institution of Oceanography La Jolla California 92093 USA; ^2^ Moss Landing Marine Laboratories Moss Landing California 95039 USA; ^3^ Institute of Marine Science University of Auckland Auckland 1142 New Zealand

**Keywords:** bivalves, bryozoans, climate, echinoderms, episodic events, filter feeders and plankton particulate size, ice, oceanography, regime shift, scaling space and time, sponges

## Abstract

Polar ecosystems are bellwether indicators of climate change and offer insights into ecological resilience. In this study, we describe contrasting responses to an apparent regime shift of two very different benthic communities in McMurdo Sound, Antarctica. We compared species‐specific patterns of benthic invertebrate abundance and size between the west (low productivity) and east (higher productivity) sides of McMurdo Sound across multiple decades (1960s–2010) to depths of 60 m. We present possible factors associated with the observed changes. A massive and unprecedented shift in sponge recruitment and growth on artificial substrata observed between the 1980s and 2010 contrasts with lack of dramatic sponge settlement and growth on natural substrata, emphasizing poorly understood sponge recruitment biology. We present observations of changes in populations of sponges, bryozoans, bivalves, and deposit‐feeding invertebrates in the natural communities on both sides of the sound. Scientific data for Antarctic benthic ecosystems are scant, but we gather multiple lines of evidence to examine possible processes in regional‐scale oceanography during the eight years in which the sea ice did not clear out of the southern portion of McMurdo Sound. We suggest that large icebergs blocked currents and advected plankton, allowed thicker multi‐year ice, and reduced light to the benthos. This, in addition to a possible increase in iron released from rapidly melting glaciers, fundamentally shifted the quantity and quality of primary production in McMurdo Sound. A hypothesized shift from large to small food particles is consistent with increased recruitment and growth of sponges on artificial substrata, filter‐feeding polychaetes, and some bryozoans, as well as reduced populations of bivalves and crinoids that favor large particles, and echinoderms *Sterechinus neumayeri* and *Odontaster validus* that predominantly feed on benthic diatoms and large phytoplankton mats that drape the seafloor after spring blooms. This response of different guilds of filter feeders to a hypothesized shift from large to small phytoplankton points to the enormous need for and potential value of holistic monitoring programs, particularly in pristine ecosystems, that could yield both fundamental ecological insights and knowledge that can be applied to critical conservation concerns as climate change continues.

Geological history and oceanographic processes are the warp and woof of the biological understanding of any marine habitat. Dayton et al. (1994)

## Introduction

As ecologists struggle to better understand resilience, there is increased interest in rare and episodic events in space and time that can have profound ecological consequences and generate legacy effects (Dayton 1989, Thrush et al. 2009). Because of the importance of episodic events, this understanding must integrate the patterns at appropriate spatial and temporal scales. Polar marine communities and their dynamics reflect both their geological histories and oceanographic climate changes, and Antarctic marine systems are particularly responsive to climate and human‐based perturbations (see reviews by Barnes and Conlan 2007, Gutt et al. 2013, 2015). McMurdo Sound is unique because it has a very sharp east‐west productivity gradient (Dayton and Oliver 1977) with another temporal productivity gradient from the 1960s and 1970s (Barry and Dayton 1988) contrasted with the 1990s–2018 (Kim et al. 2018; S. Kim, *personal communication*). In addition, the benthic communities on either side of McMurdo Sound are strongly zoned with habitats with the shallow zones <20–30 m being very different from the rest of the community. This paper addresses dramatic ecological changes that apparently began in the late 1990s in two southern McMurdo Sound benthic communities (Cummings et al. 2006, Thrush et al. 2006, Dayton et al. 2016b, Kim et al. 2018) that provide an opportunity to place the observed regime shifts into perspective.

It is now apparent that there was an important oceanographic regime shift in McMurdo Sound (southern Ross Sea) that began in the late 1990s in which sponges proliferated on artificial substrata but apparently not on the sea floor (Dayton et al. 2016b) and some filter feeder and deposit feeder populations were much reduced while others increased. These changes were associated with greatly increased turbidity (Dayton et al. 2016b). The biological changes were coincident with blocking by icebergs in McMurdo Sound starting about 2000, with several years of very heavy sea ice (Thrush and Cummings 2011, Kim et al. 2018), melting glaciers, and apparent melting of the bottom of the Ross Ice Shelf (Pritchard et al. 2012, Rignot et al. 2013). Interestingly, at the same time, the adjacent terrestrial dry valleys also experienced a strong response to a warming event that will have long‐lasting implications for both terrestrial and coastal marine ecosystems (Dayton et al. 2016a, Gooseff et al. 2017).

Large oceanographic and geological differences have resulted in contrasting benthic communities characterized by very different infaunal and epibenthic assemblages reflecting strong eutrophic and oligotrophic environments in the east and west sides of McMurdo Sound, respectively (Dayton and Oliver 1977, Barry 1988, Barry and Dayton 1988, Thrush et al. 2006). Persistence and thickness of summer ice are other important variables. The accumulation of thick, multi‐year ice on the west side rarely leaves the sound (reviewed by Kim et al. 2018). This results in strong attenuation of light affecting productivity of benthic producers and potentially reduces delivery of organic material derived from sea ice microbial production to benthic invertebrate communities, as has been observed on the east side of the sound (Dayton et al. 1986, Wing et al. 2018). These underlying variations provide a natural system in which it is possible to observe the ecological consequences of climate dynamics on divergent communities at regional and decadal scales.

The spatial and temporal changes at a regional scale across McMurdo Sound are interesting, in part, because the oceanography of the region was long thought to be highly stable and predictable (Littlepage 1965, Dayton et al. 1974), but this view was modified by observations of decadal changes (Dayton 1989, Dayton et al. 2016b, Schine et al. 2016). We focus on decadal‐scale change in major components of the two very different benthic communities. These shifts offer a unique opportunity to evaluate the ecological legacies of two different communities following a substantial climate‐driven environmental change and offer insights into the resilience of this system to future changes.

## Methods

### Large‐scale patterns among decades

**Figure 1:**
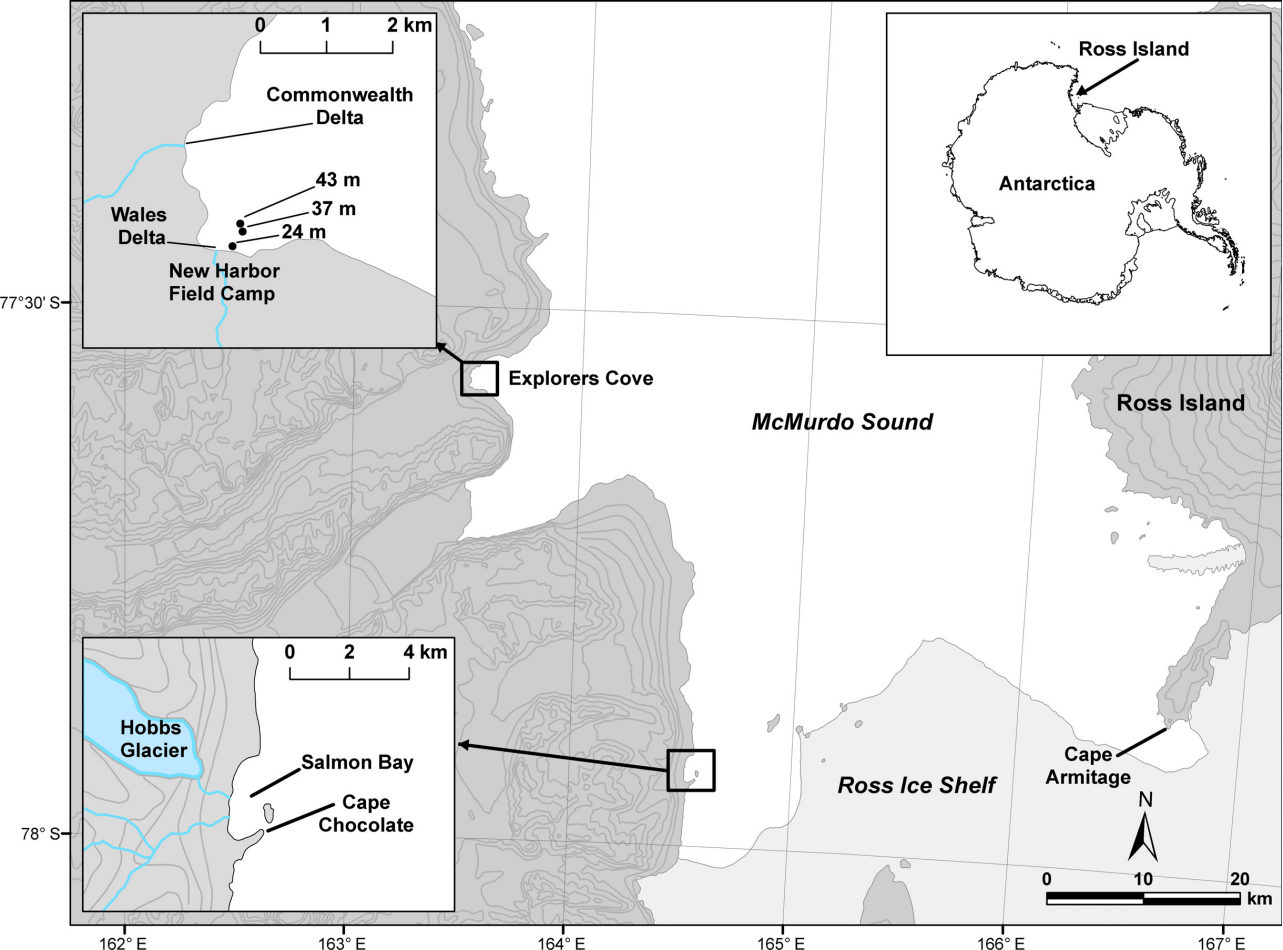
Map of the study sites in McMurdo Sound, Antarctica: Cape Armitage, Explorers Cove, and Salmon Bay.

**Figure 2:**
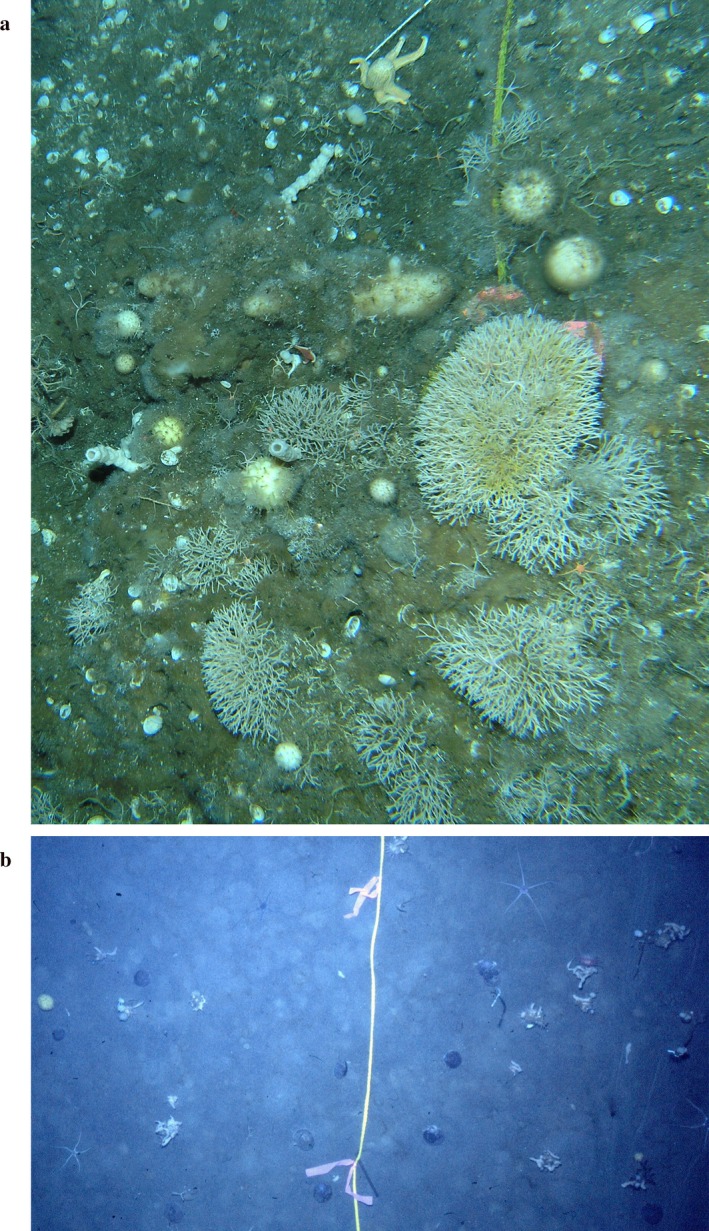
(a) Representative photo of the sponge habitat at Cape Armitage at 45 m depth. The yellow line visible at the top was placed in 1975, 35 yr previously. These transect lines were almost completely buried by the growth of the sponges and bryozoans. (b) A photo of the soft bottom habitat in Explorers Cove in 1975 at 45 m depth. The white figures are sponges being carried by the urchin *Sterechinus neumayeri*. Almost no urchins were observed in 2010.

We report samples from three locations, Cape Armitage on the east side of McMurdo Sound, and Explorers Cove and Salmon Bay on the west side of McMurdo Sound (Fig. 1). The benthic communities on each side of the Sound are known to be very different (Dayton and Oliver 1977; Fig. 2a, b).

### Cape Armitage, east side of McMurdo Sound

#### Sponge community

Data were collected from Cape Armitage sponge transects at depths of 33, 39, 42, 45, 48, and 55 m in 1968 and 2010. The 1968 taxonomy was imperfect, and in some cases what had been considered as one species actually consisted of many species that we were able to identify in 2010. In order to compare the 1968 data with the more detailed 2010 data, the more recent data were lumped as they were in 1968 (Table 1). The data for those species that were confounded in 1968 with their 2010 species designations are presented in Table 2. The most important sponge species, *Rossella podagrosa*, was misidentified as *R. racovitzae* in 1968 (Gocke et al. 2015). This is the most abundant sponge in the area, but because the individuals within patches were indistinguishable and often partially buried, the 1968 black and white photographs were inadequate and overestimated their percent cover. The 2010 ROV color photographs could not differentiate individuals, but the percent cover data were recorded. Other conspicuous Cape Armitage epifauna were areas of Bryozoa and hydroids. These species often grew in multispecies clumps and were grouped as “Bryozoa/Hydrozoa” but these patches mostly contained Bryozoa. The percent cover data were lumped into two categories, sponges and Bryozoa/Hydrozoa in order to evaluate a visually obvious shift from sponges to Bryozoa/Hydrozoa that we observed in deeper water. Percent cover data were graphed and second‐order polynomial curves were fit to the data.

**Table 1:** Mean density, size, and percent cover for the sponges and Bryozoa at Cape Armitage between depths of 30 and 58 m, for the species as they were recognized in 1968

	Density			Size			Cover		
Group/species	1968 (no./m^2^)	2010 (no./m^2^)	*P* diff	1968 (m^2^)	2010 (m^2^)	*P* diff	1968 (%)	2010 (%)	*P* diff
“Volcano” sponges	0.267 (0.03)	0.486 (0.068)	**0.0032**	0.161 (0.011)	0.063 (0.008)	**0.0003**	4.167 (0.53)	2.626 (0.417)	0.0232
“Basketball” sponges	1.822 (0.133)	1.832 (0.199)	0.9661	0.022 (0.001)	0.008 (0.001)	**<0.0001**	3.563 (0.274)	1.128 (0.121)	**<0.0001**
*Cinachyra antarctica*	1.221 (0.108)	2.769 (0.26)	**<0.0001**	0.012 (0.001)	0.004 (0.0)	**<0.0001**	1.399 (0.14)	1.052 (0.097)	0.0437
*Mycale acerata*	0.089 (0.02)	0.087 (0.022)	0.9509	0.163 (0.038)	0.057 (0.015)	0.0197	1.322 (0.373)	0.53 (0.199)	0.0621
*Polymastia invaginata*	1.252 (0.114)	1.237 (0.122)	0.9314	0.012 (0.001)	0.011 (0.001)	0.4420	1.208 (0.118)	1.026 (0.101)	0.2421
*Haliclona* spp.	2.104 (0.232)	1.391 (0.149)	0.0102	0.007 (0.001)	0.021 (0.003)	**<0.0001**	1.164 (0.167)	2.051 (0.241)	**0.0028**
*Dendrilla antarctica*	0.103 (0.024)	0.086 (0.055)	0.7705	0.051 (0.011)	0.02 (0.015)	0.0466	0.781 (0.345)	0.064 (0.043)	0.0407
*Hemigellius fimbriatus*	0.166 (0.026)	0.278 (0.07)	0.1280	0.05 (0.009)	0.024 (0.004)	0.1110	0.666 (0.141)	0.55 (0.135)	0.5524
*Sphaerotylus antarcticus*	0.228 (0.06)	0.194 (0.048)	0.6503	0.038 (0.011)	0.01 (0.002)	**0.0028**	0.643 (0.203)	0.119 (0.029)	0.0116
*Leucascus leptoraphis*	0.149 (0.021)	0.074 (0.023)	0.0151	0.018 (0.003)	0.048 (0.01)	0.0117	0.245 (0.047)	0.274 (0.084)	0.7694
Pink *Hemigellius* sp.	0.012 (0.006)	0.0 (0.0)	0.0487	0.078 (0.051)	0.0 (0.0)	NA	0.099 (0.074)	0.0 (0.0)	0.1858
*Isodictya erinacea*	0.087 (0.026)	0.089 (0.034)	0.9659	0.012 (0.002)	0.007 (0.002)	0.0662	0.097 (0.033)	0.055 (0.022)	0.2864
*Calyx* spp.	0.033 (0.014)	0.098 (0.03)	0.0512	0.013 (0.002)	0.014 (0.006)	0.8361	0.042 (0.017)	0.129 (0.06)	0.1640
*Isodictya setifera*	0.011 (0.005)	0.016 (0.009)	0.6040	0.021 (0.006)	0.008 (0.002)	0.1061	0.024 (0.013)	0.013 (0.008)	0.4664
*Kirkpatrickia coulmani*	0.018 (0.007)	0.01 (0.008)	0.4336	0.012 (0.003)	0.011 (0.001)	0.7659	0.021 (0.01)	0.01 (0.008)	0.3841
*Kirkpatrickia variolosa*	0.01 (0.005)	0.007 (0.005)	0.7072	0.018 (0.004)	0.027 (0.014)	0.6413	0.018 (0.01)	0.016 (0.011)	0.8797
*Microxina* sp.	0.014 (0.009)	0.004 (0.004)	0.1154	0.005 (0.002)	0.01 (0.0)	NA	0.008 (0.006)	0.004 (0.004)	0.2033
Bryozoa/Hydrozoa patch	NA	NA	NA	NA	NA	NA	6.885 (0.636)	28.416 (1.884)	**0.0009**

*Notes*

135 photos were analyzed for 1968 and 114 for 2010. Values and means with SE in parentheses. Also shown are *P* values for significance tests of differences (diff) between 1968 and 2010 for density, size, and cover of each species, with significant differences shown in bold font. Critical *P* values for significance were adjusted by Holm‐Bonferroni correction to account for multiple tests. “NA” refers to lack of data for colonial species or significance tests that could not be evaluated.

**Table 2:** Separation of lumped taxonomic groupings from 1968

2010 organisms included in grouping	Density (no./m^2^)	Size (m^2^)	Cover (%)
“Basketball” sponges			
“Eyeball” sponge	0.116 (0.029)	0.005 (0.001)	0.052 (0.014)
*Cinachyra barbata*	0.295 (0.056)	0.014 (0.002)	0.34 (0.075)
*Antarctotetilla leptoderma*	1.014 (0.148)	0.006 (0.001)	0.43 (0.071)
*Antarctotetilla grandis*	0.341 (0.085)	0.007 (0.001)	0.214 (0.058)
*Haliclona* spp.			
*Haliclona dancoi*	0.86 (0.106)	0.015 (0.003)	1.018 (0.176)
*Haliclona scotti*	0.536 (0.088)	0.029 (0.005)	1.049 (0.183)
“Volcano” sponges			
*Anoxycalyx joubini*	0.018 (0.013)	0.119 (0.07)	0.12 (0.076)
*Rossella antarctica*	0.087 (0.023)	0.045 (0.011)	0.316 (0.094)
*Rossella levis*	0.077 (0.029)	0.028 (0.007)	0.19 (0.08)
*Rossella racovitzae*	0.089 (0.023)	0.037 (0.01)	0.302 (0.103)
Unidentified smooth volcano, *Rossella* sp.	0.219 (0.06)	0.096 (0.012)	1.721 (0.418)

*Note*

Values are means, with SE in parentheses, for the sponges identified at Cape Armitage between depths of 30 and 58 m in 2010.

Initial sampling at the east sound, Cape Armitage, in 1968 was by scuba divers photographing transect lines marked at 1‐m intervals and imagery was analyzed with a planimeter (see Dayton et al. 1974). These data were recorded as density, size, and percent cover. In 2010, a remotely operated vehicle (ROV) capable of seafloor video surveys under ice and equipped with scaling lasers was used to revisit the 1968 transects at Cape Armitage. Most of the transect lines from 1968 were buried, but end posts and fragments of the lines were visible and for the most part the 2010 ROV surveys duplicated the 1968 diver photographs. Benthic photographic surveys conducted ancillary to other projects were run by scuba divers at Explorers Cove and Salmon Bay in 1974–1977 along transect lines marked at 1‐m intervals. Surveys were conducted by ROV in 2010, with smaller ROV photographs taken at random positions determined by timed intervals along the same depth profiles very close to the 1974–1977 transects. These surveys were usually done in the immediate area of the older transects that were partially obscured by overgrowth or sedimentation. The Explorers Cove and Salmon Bay photographs and all the ROV images were analyzed for densities of visible macroinvertebrates with ImageJ imaging software (Schneider et al. 2012). Most identifications followed Peter Bruggeman's field guide (*available online*).^1^ We conducted *t* tests to assess differences between 1968 and 2010 density, size, and percent cover of individual sponge species. Critical *P* values for inferring significance were adjusted to account for multiple *t* tests across species using the sequential Holm‐Bonferroni correction (Holm 1979).

#### Succession on rocky substratum

To study potential successional processes, a relatively flat 2 × 2 m area along a vertical rocky wall at a depth of 32 m at Cape Armitage was carefully scraped clean in October 1967. There was virtually no recruitment throughout the 1970s and 1980s, but small sponges were recorded photographically in 2010.

#### Echinoderm densities

We recorded densities of three echinoderm species at a 17 m Cape Armitage transect because the densities were obviously different in 2010. In 1967, we recorded echinoderm counts from quadrat drops along a permanent transect line and, in 2010, we surveyed the same line photographically.

### Explorers Cove, west side of McMurdo Sound

**Figure 3:**
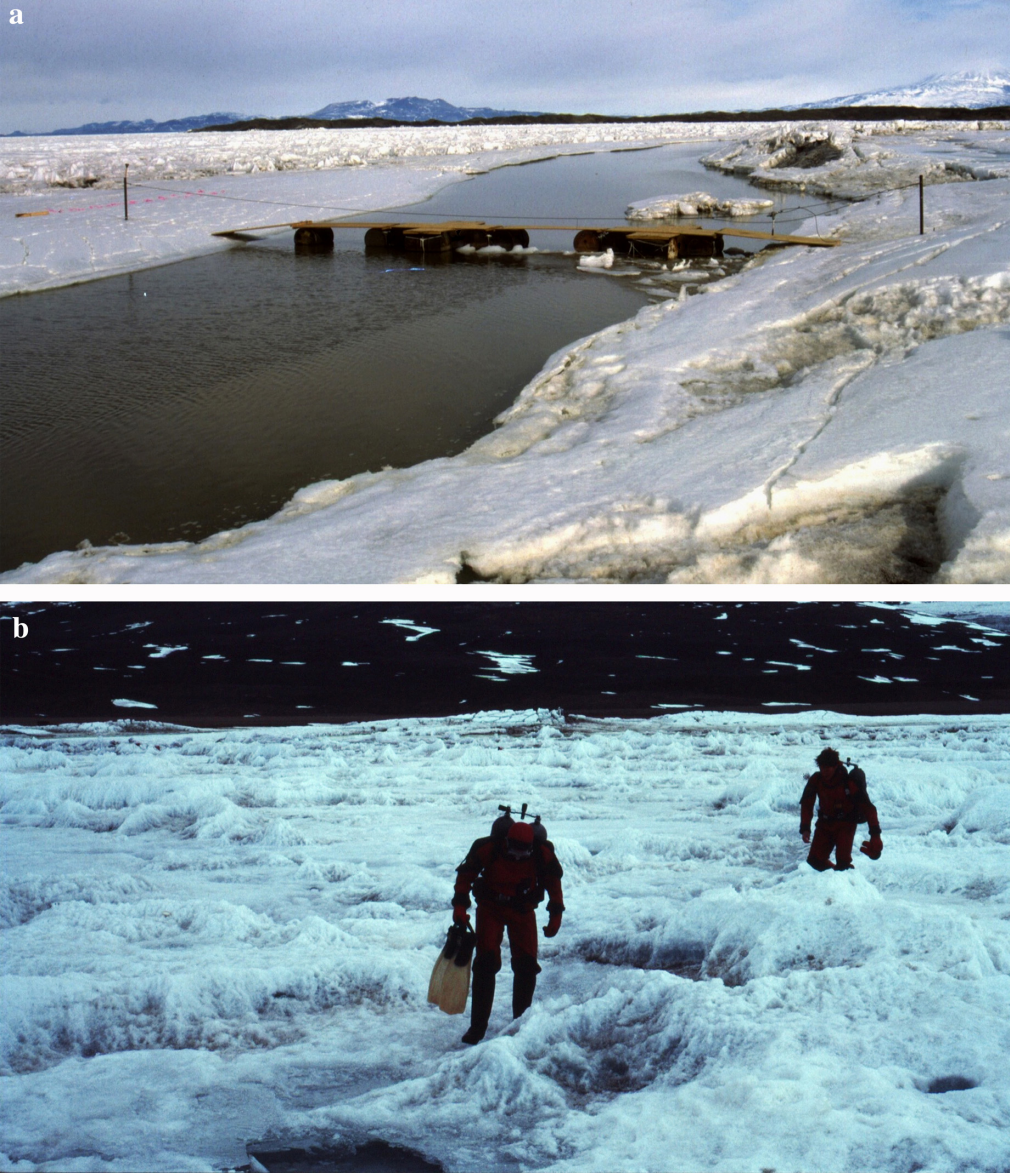
(a) Photo of the brackish moat at Salmon Bay with the ice wall on the left. The sea ice floats up during high tides allowing the productive water in the moat to escape over the ice wall into the study site. The “bridge” was designed to allow divers and tenders to access the dive site from shore. (b) Divers at Salmon Bay accessing the dive hole over difficult multi‐year sea ice characterized by layers of windblown gravel that absorbs solar energy and melts about 1 m into the ice, leaving a pool of water covered with thin ice.

Explorers Cove is a soft bottom community with very little epifauna (see Fig. 2b). There are two drainages from nearby glaciers; the Wales Glacier on the south drains into the area adjacent to our long‐term study location. The width of the delta grew from about 3 to >30 m between 1974 and 2010, but the amount of sedimentation along our transect lines was <5 mm in the almost 40 yr (Walker et al. 2013; P. K. Dayton, *personal observation*). The cove is characterized by thick (usually 4–7 m) sea ice that rarely goes out to sea, and the shoreline is defined by an ice wall at about 5 m depth; this barrier separates the summer runoff from the sea (see Stockton 1983). The brackish water in the moat impounded behind the ice wall (Fig. 3a) is rich in nutrients (Mikucki et al. 2015) and can be relatively warm and very productive. During spring high tides, the sea ice floats higher than the ice wall, allowing the cold, dense, sea water to flow into the moat where it sinks, pushing the brackish water with streaming algal material over the edge of the ice wall. This sinks to the bottom, resulting in a band of productivity that is fed on by dense populations of the Antarctic scallop, *Adamussium colbecki*. The bottom adjacent to the ice wall is sandy but quickly grades into a silty mud. Because of the periodic input of organic material from the moats, the bottoms along the west sound out to 100–200 m probably are exposed to more primary production that those farther offshore. The soft bottom habitats along the west side of the sound are further described in Dayton and Oliver (1977) and Stockton (1983, 1984). There was very little epifauna (see Fig. 2). Here, we report and contrast the densities of the 11 most abundant species from 1974 to 1977 and 2010 to depths >40 m.

### Salmon Bay, west side of McMurdo Sound

The Salmon Bay (Fig. 1) location differs from Explorers Cove as it is farther south and protected by Cape Chocolate. The protected nature of the site reduces the probability of the sea ice breaking up and going out, resulting in thick ice with layers of gravel frozen into the 7–8 m thick sea ice (Fig. 3b). We believe it may not have gone out between the turn of century “heroic age” and 2011 and 2014 when it did go out (S. Bowser, *personal communication*). Sites at 18 and 21 m depth were sampled in 1988 and 1989 by divers and in 2010 by ROV. The very different biota at Salmon Bay was virtually obliterated by a massive flood and sedimentation event around 2001 (Dayton et al. 2016a). We combined the workable photographs from 1988 to compare them with the ROV photographs taken in 2010. We were able to locate the exact sites of the 1988–1989 data because the sea ice had not gone out in the intervening years and our old dive holes were visible as clear blue ice in otherwise broken and rotten ice. Thus, although the bottom was completely different, the 2010 samples were in the same place. At Salmon Bay, additional depths of 12, 25, 28, and 31 m were sampled in 2010 by ROV.

## Results

### Large‐scale patterns among decades

There are large differences in invertebrate abundance across McMurdo Sound reflecting the oceanographic patterns (Thrush et al. 2006). We present a summary of spatial patterns across McMurdo Sound, as well as changes between decades comparing patterns in the 1960s and 1970s with 2010. We report changes in invertebrate abundances on both sides of the sound and these appear to reflect major oceanographic changes described in Kim et al. (2018). In addition, there is important spatial and temporal variability at smaller scales, particularly considering species‐specific patterns within sites. Below, we present the species‐specific data among sites, as these are important to understanding more fully the patterns and variability observed.

### Cape Armitage, east side of McMurdo Sound

#### Sponge community

We hypothesized (Dayton et al. 2016b) that the natural sponge community did not reflect the massive recruitment and growth of many species of sponges on artificial substrata. Here we evaluate changes by comparing data from Cape Armitage collected and analyzed in 1968 with similar data from 2010. Sponge density, mean size, and percent cover at depths of 30–58 m at Cape Armitage for 1968 and 2010 are shown in Table 1. The three categories presented represent different types of information. The density data relate to the population demographics of each species, and the mean individual size may represent relative ages, also of demographic interest. Finally, the percent cover of each species refers to space occupancy and represents information relevant to the ecosystem/functional sense of community dynamics. Table 1 presents mean density, size, and percent cover of sponges at McMurdo Station in 1968 and 2010 along with probability values and results of significance testing for *t* tests of the differences between time periods for each of the species indicated by bold face in Table 1. While most of the species showed no differences, some did. For example, there were more “volcano sponges” and *Cinachyra antarctica*, but the individuals were smaller and had a smaller percent cover. This observation is consistent with high mortality and recruitment of *Anoxycalyx joubini* (Dayton et al. 2016) and a surprisingly high recruitment of *Cinachyra* with more, smaller, individuals. There were fewer but larger *Haliclona*, suggesting some mortality and higher growth from a species known to grow relatively quickly. With the exception of *Haliclona* spp., these sponges were not important components of the recruitment event discussed in Dayton et al. (2016b). For this reason, a general null hypothesis of no discernable change in the natural sponge community across decades would appear to hold for most of the sponge species.

Large patches of the enigmatic *Rossella podagrosa* are difficult to measure and evaluate as many are buried in the spicule mat, and most have small buds attached to larger ones (see Gocke et al. 2015). Most of the transect lines at Cape Armitage were overgrown and many large patches of *R. podagrosa* were seen overgrowing transect lines in the 2010 photos (see Fig. 2) such that the population appeared very dynamic. Unfortunately, the poor quality black and white photos of 1968 did not lend themselves to accurate density estimates, so a quantitative comparison was not possible. *R. podagrosa* was relatively abundant with 9.9% cover overall at Cape Armitage sites in 2010. Table 1 presents the same taxonomic units used in the 1974 paper and Table 2 presents the more detailed 2010 data for the individual species that were lumped in Table 1.

**Figure 4:**
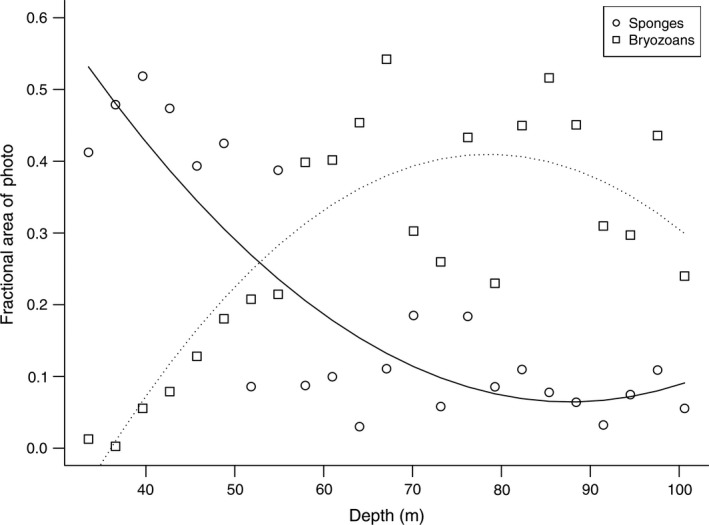
Percent cover of all sponges vs. Bryozoa/Hydrozoa patches. Curves indicate second‐order polynomial fits to the data.

Earlier (Dayton et al. 1974) we observed that total sponge cover declined with increasing depth while Bryozoa and Hydrozoa increased with depth. Our comparison of the depth patterns of total sponges and Bryozoa/Hydrozoa for the 2010 data demonstrate a marked shift in the percent cover of sponges vs. Bryozoa. The mean percent cover of sponges declines precipitously with depth from about 50% at 40 m to about 10% at 50 m. The mean percent cover of Bryozoa increases from 0% cover at 30 m to about 40% at 60 m with a high of 54% at 70 m depth. Fig. 4 shows the relatively distinct cross over point at about 54 m with the pattern consistent to 100 m.

#### Succession on rocky substratum

The 1967 effort to evaluate succession on the cleared area of the Cape Armitage wall recorded no recruitment at all the following year. The cleared area was followed opportunistically throughout the 1970s with only a few hydroids appearing but, in the late 1980s, some small *Polymastia invaginata* appeared. By 2010, there was intense recruitment of *P. invaginata* (33 individuals/m^2^), *Inflatella belli* (8 individuals/m^2^), *Sphaerotylus antarcticus* (8 individuals/m^2^), and *Suberites caminatus* (14 individuals/m^2^). There were a few scattered Bryozoa and one each of *Artemidactis victrix*,* Stomphia selaginella*,* Odontaster validus*, and *O. meridionalis*. Almost 70% of the total surface area was unoccupied by invertebrates, suggesting that competition for primary space was not a factor in this habitat for over 40 yr (Table 3).

**Table 3:** Recruitment of sponges and Bryozoa observed in 2010 on rocky wall at 32 m depth at Cape Armitage that was cleared in October 1967

Organism	Density (no./m^2^)	Size (m^2^)†	Cover (%)
*Polymastia invaginata*	33.18	0.007 (0.0043)	22.32
*Inflatella belli*	7.54	0.004 (0.0026)	2.94
*Suberites caminatus*	13.58	0.002 (0.0014)	2.63
*Sphaerotylus antarcticus*	8.29	0.005 (0.0027)	3.99
Bryozoa	3.77	0.006 (0.0023)	2.41

*Note*

The clearing was ~4 m^2^ but only the center 1.3 m^2^ was analyzed.

† Means with SE in parentheses.

#### Echinoderm densities

Responding to a conspicuous decrease in *Odontaster validus* coincident with the hypothesized decrease in large phytoplankton and benthic productivity, we resurveyed a 1967 transect in 2010 at 17 m to record the densities of *O. validus*,* Sterechinus neumayeri*, and *Diplasterias brucei* directly or indirectly influenced by deposition of large phytoplankton and benthic productivity in the only area with sufficient data for such a comparison (Table 4). The *O. validus* and *S. neumayeri* primarily subsist on benthic diatoms and phytoplankton detritus (Pearse 1965, Pearse and Giese 1966) and, in this area, *D. brucei* specializes on the filter‐feeding bivalve *Limatula hodgsoni* (Dayton et al. 1970). The reduction in their density is consistent with the hypothesized oceanographic shifts discussed in Thrush and Cummings (2011).

**Table 4:** Density of echinoderms *Odontaster validus*,* Sterechinus neumayeri*, and *Diplasterias brucei* in 1967 and 2010 at 17 m

Organism	Density (no./m^2^)	
	1967	2010
*Diplasterias brucei*	0.399 (0.126)	0.071 (0.0552)
*Odontaster validus*	12.997 (2.886)	2.802 (0.4823)
*Sterechinus neumayeri*	1.025 (0.353)	0.329 (0.137)

*Note*

Values are means with SE in parentheses.

### Explorers Cove, west side of McMurdo Sound

**Figure 5:**
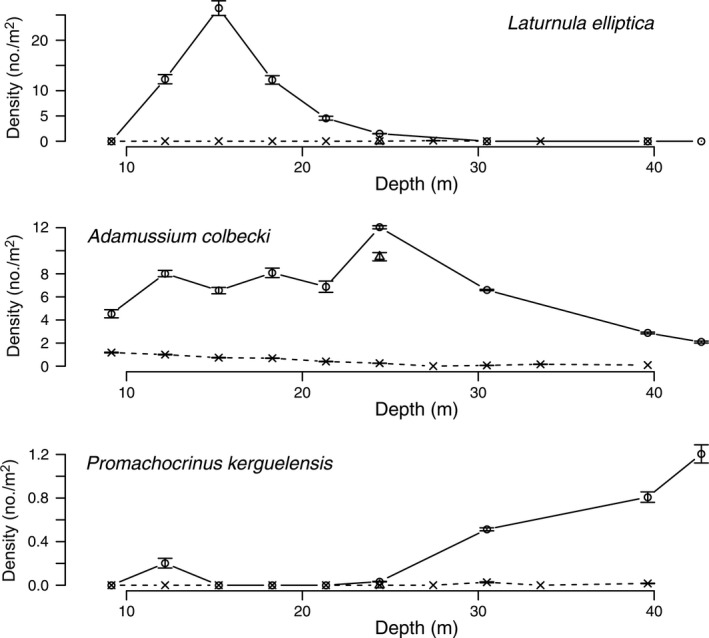
Mean densities of bivalves *Laternula elliptica*,* Adamussium colbecki*, and the crinoid *Promachocrinus kerguelensis* along the depth gradient at Explorers Cove in 1974–1977, a transect for *Adamussium* in 1985, and 2010. The circles and solid lines represent pooled data from 1974 to 1979 and × and dashed lines represent data from 2010. The triangles represent poor photo transects for 1985. The error bars represent standard error.

**Figure 6:**
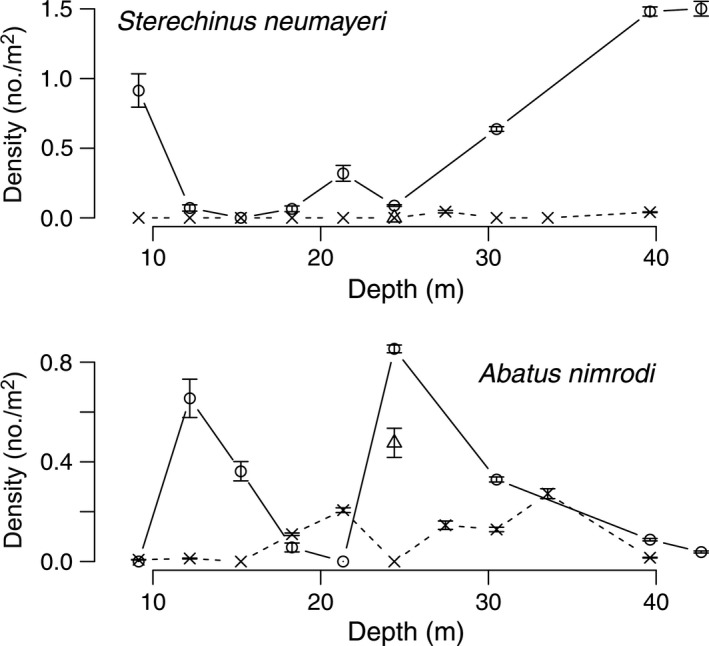
Mean densities of the deposit‐feeding sea urchin *Sterechinus neumayeri* and the heart urchin *Abatus nimrodi* across the depth gradients at Explorers Cove. The circles and solid lines represent pooled data from 1974 to 1977 and x and dashed lines represent data from 2010. The triangles represent poor photo transects from 1985. The error bars represent standard error.

**Figure 7:**
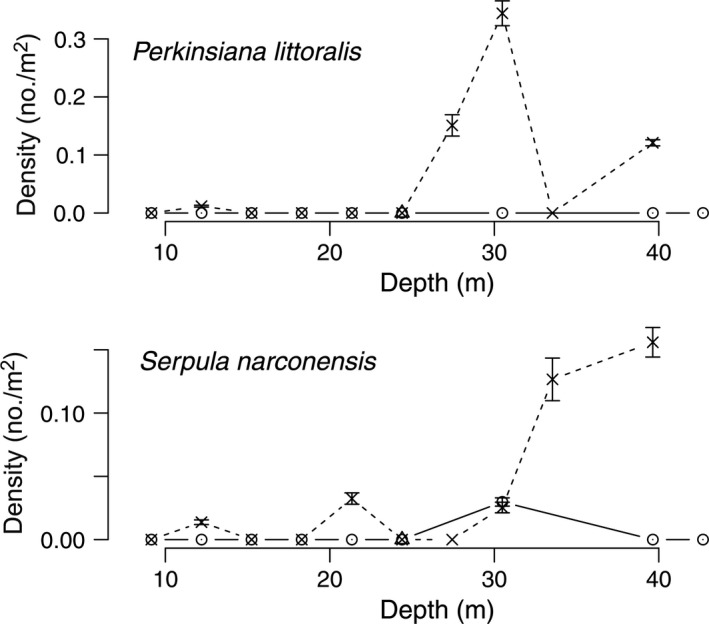
Mean densities of the polychaetes *Perkinsiana littoralis* and *Serpula narconensis* across the depth gradients at Explorers Cove in the 1970s and 2010. The circles and dashed lines represent pooled data from 1974 to 1979 and × and dashed lines represent data from 2010. The error bars represent standard error.

**Figure 8:**
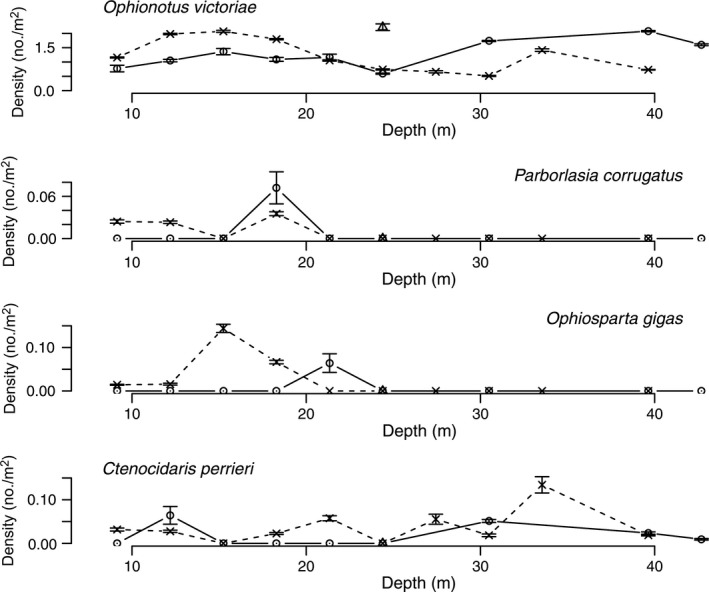
Mean densities of predators (*Ophionotus victoriae*,* Parborlasia corrugatus*,* Ophiosparta gigas*, and *Ctenocidaris perrieri*) at Explorers Cove, New Harbor with the data for 1974–1979 summed. The circles and dashed lines represent pooled data from 1974–1979 and × and dashed lines represent data from 2010. The triangles represent poor photo transects for 1985. The error bars represent standard error.

Diver surveys from 1974 to 1977 at Explorers Cover were repeated in 2010 with the ROV to evaluate potential changes resulting from the shifts in the oceanographic regime. Densities of suspension‐feeding invertebrates including bivalves and a crinoid (Fig. 5), deposit feeders (Fig. 6), polychaetes (Fig. 7), and carnivores (Fig. 8) show depth specific changes between these time periods.

In the 1970s, the large clam, *Laternula elliptica*, had a peak abundance between 12 and 27 m (Fig. 5), but it disappeared completely by 2010. Lohrer et al. (2013) reported that there were no *L. elliptica* in the vicinity of our sites in 2009. The decline in deeper water may reflect the fact that the shallow sites have more benthic productivity and food, reflecting the spillover from the moat, the enhanced light along the tide cracks, and because there seemed to be somewhat stronger currents along the edge of the cove exposing filter feeders to more food (P. K. Dayton and S. F. Thrush, *personal observations*).

The scallop, *Adamussium colbecki* had relatively high densities in the 1970s, especially at 27 m. But it is important to note that these data do not include the extremely high densities (>100 individuals/m^2^) Stockton (1983) reported along the ice wall because our long‐term study sites were away from the ice wall. The single datum from 1985 was relatively high at the 27‐m site, implying that the dramatic population crash observed (Fig. 5) in 2010 began after 1985.

The common Antarctic crinoid, *Promachocrinus kerkuelensis*, is a filter feeder usually perched on sponges or boulders or our artificial structures where it has better access to the very weak currents that characterize Explorers Cove. Thus, density data are highly dependent upon the foundational habitat structures. In this sense, our data are not strictly comparable but probably reasonably meaningful because the 1970 era data were collected along the same transect lines with the same scattered structures (rocks and sponges), and the 2010 ROV transects followed the old lines or at least were very close to them. The collapse of the crinoid density (Fig. 5) in 2010 does not reflect a loss of sponges as they were at approximately the same density on the bottom despite the strong sponge recruitment on artificial structures reported in Dayton et al. (2016b).

The sea urchin, *Sterechinus neumayeri*, was abundant between 1974 and 1977 in the shallow water area with its higher benthic productivity, declined at 12–18 m, but then became very abundant at 30–40 m depths (Fig. 6). However, their density also fell precipitously by 2010. The heart urchin, *Abatus nimrodi*, was relatively rare but usually obvious in the photographs. *Abatus nimrodi* has a patchy distribution but its densities were much lower in 2010. And as with *A. colbecki*, the 1985 mean was relatively high, again suggesting a post‐1985 density reduction.

*Gersemia antarctica* is a large (up to 4 m tall in rare cases) nephtheid alcyonacean soft coral that was common at Explorers Cove in the 1970s. This large soft coral was patchy but abundant in all our areas but more common with fine sediment where it is best able to use the unique deposit‐feeding behavior described by Slattery et al. (1997) in which it flops down on the bottom and consumes organic material, moving to a new area after scouring the adjacent bottom. In areas near the two drainage deltas in Explorers Cove we estimated densities approaching 1 individual/10 m in the 1970s, and Slattery et al. (1997, Slattery and McClintock 1995) surveyed several areas in the late 1980s and early 1990s and reported densities in the range of 4 individuals/100 m. When we returned in 2010, *G. antarctica* was almost completely absent as we found <10 over a very large search area. In contrast, they were never seen on the east side of the sound in the 1970s–1990, but in 2010 there was a very similar alcyonacean on hard rather than soft substrata at the McMurdo Station jetty and at Evans Wall in the east sound. Unpublished genetic analysis (H. Cha, *personal communication*) suggests that this is also *G. antarctica,* occupying a very different habitat.

In contrast, the abundance of the sabellid polychaete, *Perkinsiana littoralis*, follows the opposite temporal pattern (Fig. 7); it was never recorded on the 1970s transects but had a strong abundance peak in the deeper water in 2010. Other photographs show that they did occur very rarely at Explorers Cove in the 1970s, although virtually none were in our transects, so the spike at 27 and 30 m represents a real change. In the same way, the serpulid polychaete, *Serpula narconensis*, was rare in the 1970 era, but occurred in relatively high numbers in deeper water (27–40 m) in 2010 (Fig. 7).

The data for carnivorous species show no clear patterns between the 1970s and 2010 (Fig. 8). These species are highly mobile and aggregate on patches of prey. *Ophionotus victoriae*, the very common brittle star, is a carnivore but apparently is able to subsist on detritus (Norkko et al. 2007, Lohrer et al. 2013). It is patchy on several scales, and our data do not appear to show a trend. *Parborlasia corrugatus*,* Ophiosparte gigas*, and *Ctenocidaris perrieri* are particularly active, patchy, and sufficiently rare at Explorers Cove that the data in Fig. 8 do not indicate change between the two sampling periods. The peak in the pencil urchin, *C. perrieri*, may reflect the fact that transects were in the vicinity of the floating settling plates laden with large sponges (Dayton et al. 2016b) that were sinking where *C. perrieri* and *Sterechinus neumayeri* quickly congregated to scavenge the sunken material.

### Salmon Bay, west side of McMurdo Sound

The benthic habitat in Salmon Bay had been subjected to a massive flood in about 2001 (Dayton et al. 2016a) that covered the bottom with >50 cm of sediment. For this reason the Salmon Bay epifaunal density data compare two very different situations. The data from 1988–1989 reflect a mature benthic community far from the major sources of pelagic production (Barry 1988). The ice had not broken out between 1988 and 2010 so our data represent a unique observation of invertebrate settlement following a disturbance in a habitat relatively far from the open water subject to the northern flow of nutrient‐poor water from under the Ross Ice Shelf (Dayton and Oliver 1977, Barry 1988, Barry and Dayton 1988). Assuming that the flood sedimentation eliminated the animals observed in 1988, most of the species observed in 2010 reflect dispersal into the area.

**Figure 9:**
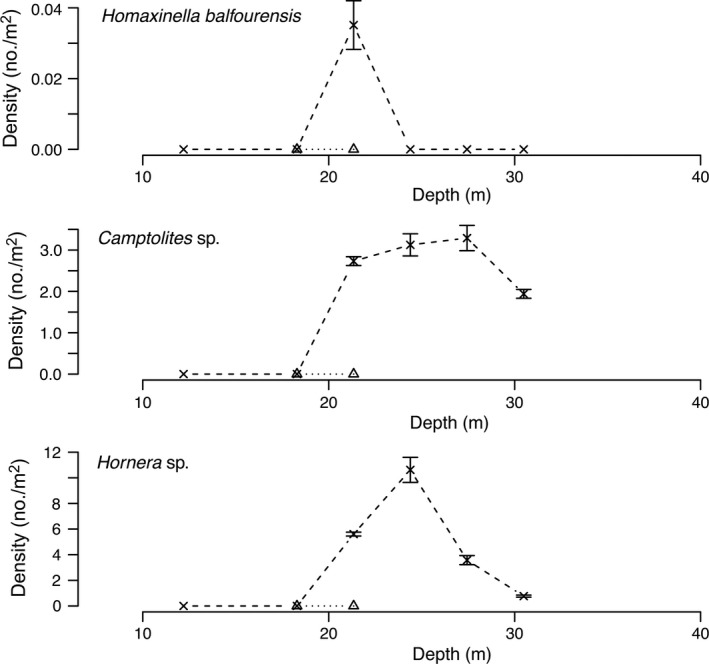
Mean density of an opportunistic sponge, *Homaxinella balfourensis*, and two unidentified Bryozoa, *Camptolites* sp. and *Hornera* sp. The × and dashed lines represent data from 2010 and triangles and dotted lines represent the lack of individuals in 1988. The error bars represent standard error.

*Homaxinella balfourensis*, a common sponge known for episodic settlement (Dayton 1989), was not seen in 1988, but had a spike at 21 m in 2010 (Fig. 9). By far the most abundant recruits were two species of Bryozoa tentatively identified as species of *Camptoplites* sp. and *Hornera* sp. These species were not uncommon at Explorers Cove and were observed at Salmon Bay in the 1980s, though neither showed up in transects. However, they were very abundant in depths >20 m in 2010.

**Figure 10:**
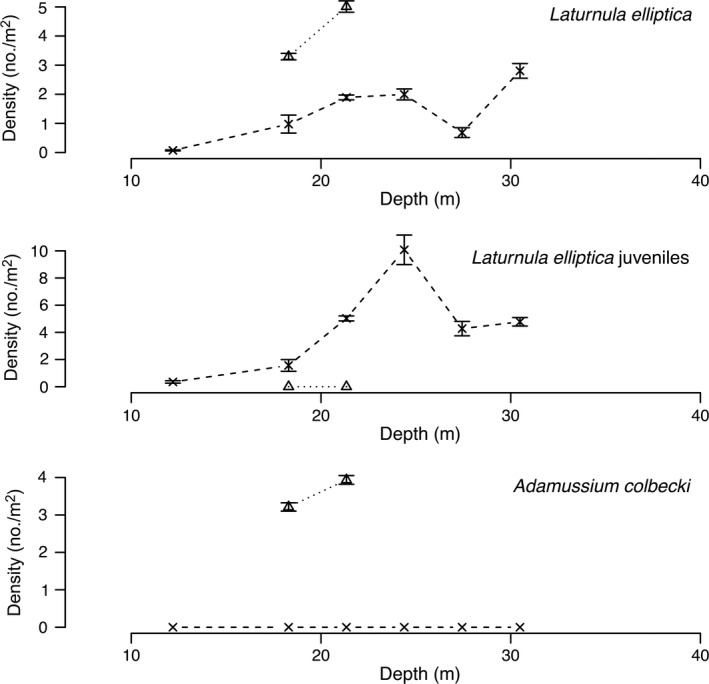
Densities of bivalves (*Laturnula elliptica* and *Adamussium colbecki*) across a depth gradient at Salmon Bay. The triangles and dotted lines represent transects in 1988 and the × and dashed lines represent data from 2010. The error bars represent standard error.

*Laternula elliptica* was extremely abundant in 1988 (Fig. 10), but it is unlikely that individuals would have survived >50 cm of sedimentation. The relatively large numbers observed in 2010 had small siphons and probably represent a strong recruitment event. We also recorded densities of “juvenile *Laternula*” that were patchy but conspicuous in some of the ROV photos, so it is apparent that in 2010 a major *L. elliptica* recruitment event was underway at Salmon Bay but not Explorers Cove. In 1988, *Adamussium colbecki* was extremely abundant near the wall, probably with densities >100 individuals/m^2^ as reported by Stockton (1983, 1984) for Explorers Cove. They also occurred at high densities at 18 and 21 m in 1988, but they were completely gone in 2010.

**Figure 11:**
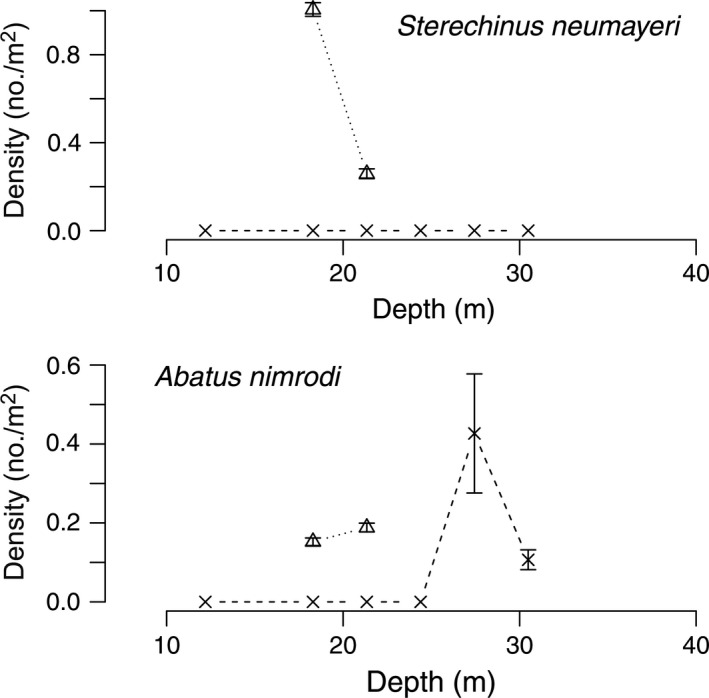
Densities of deposit feeders *Sterechinus neumayeri* and *Abatus nimrodi* across a depth gradient at Salmon Bay. The triangles and dotted lines represent transects in 1988 and the × and dashed lines represent data from 2010. The error bars represent standard error.

The urchin *Sterechinus neumayeri* was relatively abundant in 1988 at Salmon Bay but totally missing in 2010 (Fig. 11). *Abatus nimrodi* was relatively common at 18 and 21 m in 1988 but was also common in the deeper depths in 2010, either because some may have survived the sedimentation or probably reflecting recruitment after the flood.

**Figure 12:**
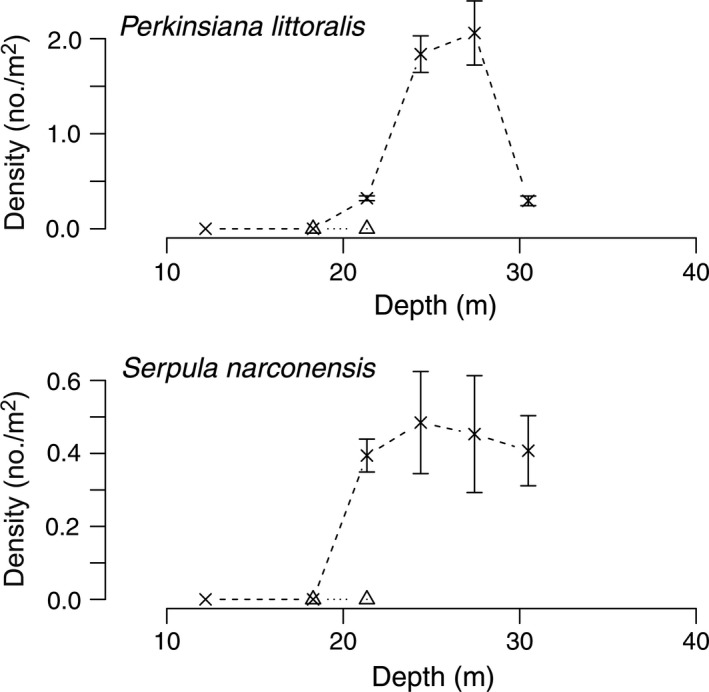
Density of polychaetes *Perkinsiana littoralis* and *Serpula narconensis* across a depth gradient at Salmon Bay. The triangles and dotted lines represent transects in 1988 and the × and dashed lines represent data from 2010. The error bars represent standard error.

*Perkinsiana littoralis* and *Serpula narconensis* were not observed in 1988–1989 in Salmon Bay but were abundant in 2010 (Fig. 12). These are the same species of polychaetes that showed up at Explorers Cove in 2010 so this recruitment event was consistent in both west sound locations.

**Figure 13:**
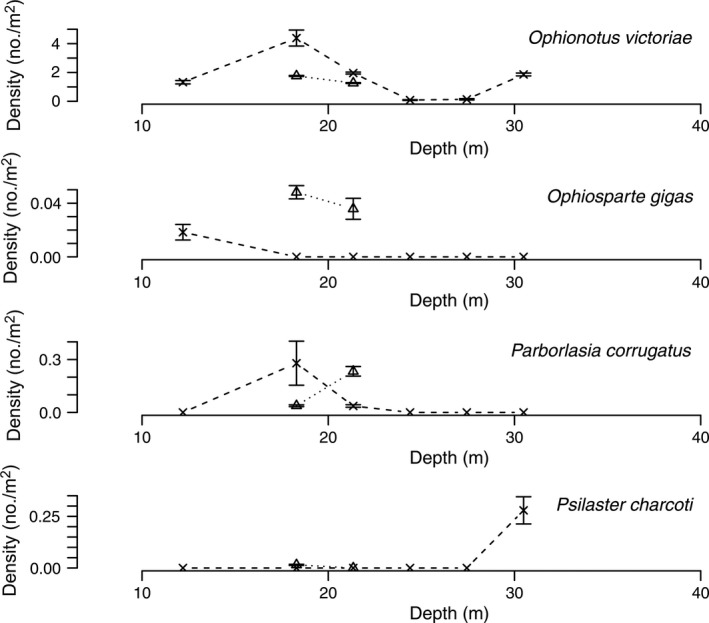
Density of carnivores (*Ophionotus victoriae*,* Ophiosparte gigas*,* Parborlasia corrugatus*, and *Psilaster charcoti*) across a depth gradient at Salmon Bay. The triangles and dotted lines represent transects in 1988 and the × and dashed lines represent data from 2010. The error bars represent standard error.

In 1988, the haphazardly dropped transects did not sample the large dense patches of *O. victoriae* that were observed. In 2010 some high‐density patches were sampled, and overall the population did not seem much different (Fig. 13). Similarly, *P. corrugatus* were more common than implied by the transect data in 1988; large aggregations were observed apparently eating *L. elliptica* but the random transects did not sample the aggregations. The 2010 data included a few *P. corrugatus*, but in the extensive ROV surveys no large aggregations were observed.

### Summarizing decadal changes

In marked contrast to the changes observed on artificial substrata (Dayton et al. 2016b), the overall sponge association on natural substrata at the east Sound site was not much changed over the decades despite some indication of species turnover (Table 1). This paradox emphasizes the importance of better understanding the recruitment dynamics of Antarctic sponges. However, marked reductions in populations of deposit‐feeding echinoderms, the alcyonacean *Gersemia antarctica*, the filter feeding crinoid *Promachocrinus kerkuelensis*, and filter feeding bivalves suggest a marked reduction in the amount of diatoms and large phytoplankton. In contrast, we observed an explosive growth of sponges on artificial substrata on both sides of McMurdo Sound (Dayton et al. 2016b) and two species of filter feeding polychaetes at Explorers Cove. Sponges and these polychaetes are known to rely on very small food particles.

Natural history observations on movement, foraging behavior, and additional information on the above observations can be found in Appendix S1.

## Discussion

Ecological resilience involves understanding both the potential perturbations and the recovery processes. It is important to focus on large‐scale processes in space and time, especially as they relate to rare and episodic events (Haury et al. 1978, Hewitt et al. 2007). The benthic communities on either side of McMurdo Sound are zoned. The Cape Armitage community on the east side of the Sound is distinguished by a shallow (to 30 m) zone characterized by disturbance and much higher productivity (Dayton et al. 1970, 1986), a sponge community (30–54 m) and a bryozoan‐based community (54–100 m, Fig. 4). The zonation at Explorers Cove is not so well defined, however it is clear that there is a very shallow band (to about 20 m) that tends to be much more productive and markedly different from 20 to 43 m. It is important that generalizations be constrained to the appropriate depth zones.

In McMurdo Sound, the perturbations involve slow responses to long‐term climate forcing or a massive episodic disturbance, while recovery reflects varying successional processes defining ecosystem resilience (Thrush et al. 2009). This paper reveals changes in regional oceanographic processes as the perturbation in the late 1990s and early 2000s. The unexpected ecological changes unprecedented over 40+ yr of observation appear to be defined by the resources available to filter‐feeding benthic animals. These species provide further habitat structure on the seabed, offering refugia from predation and sediment disruption to settlers and influence benthic‐pelagic coupling on the bed.

Changes at local scales may involve positive or negative feedbacks such that local‐scale processes build on each other, resulting in alternate stable associations (Dayton et al. 1999, Thrush et al. 2009, Thrush and Dayton 2010). Episodic events can have long‐lasting impacts on benthic systems through multiple pathways that may include founder effects (Thorson 1966), various feedback loops such as Allee effects (Bruno et al. 2003), and subsequent community development. Such local and large‐scale processes may induce cumulative changes that drive ecosystems into more stable phase shifts (Pauly 1995, Thrush et al. 2009). In Antarctica, decadal regime shifts include small but important variations in temperature associated with the formation of frazil ice and anchor ice disturbance (See Barry and Dayton 1988, Dayton 1989), intensity of iceberg grounding (Gutt 2001, Thrush et al. 2006), and sea ice cover, driving regional changes in productivity (Loeb et al. 1997, Ainley et al. 2005, Gutt et al. 2013, Kim et al. 2018). There is a significant need to better understand episodic events and decadal shifts, especially in relation to the impacts of a warming climate in polar regions.

The coupling of oceanographic productivity with benthic communities is well known, and the McMurdo Sound system receives most of its net primary productivity from the large highly productive Ross Sea polynya (Grebmeier and Barry 1991, Dayton et al. 1994, Barry et al. 2003). There is also thought to be a smaller input to the benthic invertebrate community of organic material from sea ice microbial production that also changed with changes in sea ice conditions between 2008 and 2014 (Wing et al. 2018). The correlation between primary and benthic production is common in the oceanographic literature, but rarely considered in terms of such strong gradients in time and space and the now recognized changes in sea ice conditions at annual to decadal scales. This paper, with the previous papers on sponge recruitment (Dayton et al. 2013, 2016b) documents very significant changes in several populations of benthic organisms from the 1980s to 2010. Here, we consider the potential causes of this major regime shift in benthic communities in McMurdo Sound.

The shallow water benthic community of McMurdo Sound, Antarctica has remarkably strong east‐west and north‐south gradients reflecting the availability of primary productivity and sea ice persistence (Dayton and Oliver 1977, Dayton et al. 1986, Barry and Dayton 1988, Thrush et al. 2006, Conlan et al. 2010, Kim et al. 2018). Starting sometime in the late 1990s, there were dramatic ecological changes in the southern parts of the Sound (Dayton et al. 2016b). We compare benthic epifauna on both sides of the McMurdo Sound from the 1960s through the 1980s with similar data from 2010 in an effort to deduce the processes resulting in the massive sponge recruitment on artificial substrata (Dayton et al. 2013, 2016b) and the epifaunal responses observed in this paper. The large icebergs that grounded along the northern part of McMurdo Sound had significant effects on the McMurdo Sound ecosystem (Seibel and Dierssen 2003, Conlan et al. 2010, Thrush and Cummings 2011). Kim et al. (2018) report thicker ice than usual in 1995 and 2000–2004 when the sound exit was blocked by mega‐icebergs, but otherwise the sea ice within McMurdo Sound has been relatively constant since the late 1950s. Finally, potentially enhanced iron input (Hawkings et al. 2014) from increased melt of glaciers and ice shelves (Pritchard et al. 2012, Rignot et al. 2013) is another factor that may contribute to the changes. We evaluate these hypothesized factors and suggest that the feeding biology of the species demonstrating population changes reflect shifts within the plankton, not necessarily in overall productivity, but possibly a shift to significantly smaller cells of planktonic primary producers.

The dramatic recruitment of sponges on artificial structures on both sides of the sound (Dayton et al. 2016b) reflects an important environmental change resulting in sponge recruitment success via feeding biology, because sponges retain only very small particles of bacteria and protists (Reiswig 1990, Orejas et al. 2000, Leys et al. 2007, Thurber 2007, Yahel et al. 2007). It is interesting to note that the natural benthic changes do not reflect the massive sponge recruitment seen on artificial substrata, and the data in Table 1 show that sponge species respond in different ways. Those sponge species that exhibited recruitment on the natural bottom and the cleared rocky wall (*Polymastia invaginata*,* Inflatella belli*,* Sphaerotylus antarcticus*, and *Suberites caminatus*) did not recruit on the artificial substrata. Indeed, *I. belli* and *S. caminatus* that were conspicuous recruits on the cleared wall were not recorded from the transects (Table 1). It is obvious that sponges have very different “niches” (c.f. Thurber 2007) and are complicated animals in need of much more research. However, the fact that the 1974 transect lines at Cape Armitage were mostly buried by sponges that recruited since at least the 1980s suggests that there has been considerable if unmeasured sponge turnover.

The natural sponge association reported in Table 1 exhibited relatively little change over four decades despite the fact that there was a massive recruitment event on artificial substrata as well as significant recruitment on the rocky wall. This suggests a high degree of resilience, or resistance to disturbance, in the natural sponge association. Two processes appear to be involved. First, we speculate that the recruitment and growth of the sponges on the artificial substrata (Dayton et al. 2016b) relates to a shift in food availability in which the plankton shifts from large to the very small particles that the sponges utilize. But why did the abundance of sponge propagules reflected in the artificial substrata and rocky wall recruitment have such limited recruitment success in the natural community? We speculate that the resistance to change in the natural community reflects very effective predation on the settling propagules, possibly involving Foraminifera (Suhr et al. 2008), amphipod crustaceans (Oliver et al. 1982, Oliver and Slattery 1985), as well as many other benthic predators such as Platyhelminthes, ophiuroids, cnidaria, and polychaetes.

The echinoderms *Odontaster validus* and *Sterechinus neumayeri* generally have very broad diets (Norkko et al. 2007), but at Cape Armitage they derived most of their nutrition from benthic diatoms and plant debris (Pearse 1965, Pearse and Giese 1966, Dayton et al. 1974, McClintock 1994) and *Diplasterias brucei* almost exclusively consumed the filter feeding bivalve *Limatula hodgsoni* in this area (Dayton et al. 1970). We were not able to visually quantify the bivalve *L. hodgsoni* because it burrows into the spicule mat; however, there were relatively few dead shells in the 1960s‐era photographs and an impressive number of shells in most of the 2010 pictures, suggesting a large mortality event for this filter‐feeding clam congruent with bivalves in the west Sound. Table 4 demonstrates declines in the three echinoderm species, an observation consistent with the hypothesized decline in large phytoplankton.

In contrast to the limited changes at Cape Armitage, there were marked changes in the abundant benthic species on the west side of the sound at Explorers Cove. In addition to the recruitment of sponges on artificial substrata (Dayton et al. 2016b), we saw marked declines of the filter feeders *L. elliptica*,* A. colbecki*, and *Promachocrinus kerguelensis* as well as a marked increase in two species of filter‐feeding polychaetes, *Perkinsiana littoralis* and *Serpula narconensis*. Bivalves are known to rely on large food particles (Ward and Shumway 2004), and the collapse of *L. elliptica* and *A. colbecki* may reflect a shift from large to small phytoplankton that would also explain the increase in the polychaetes that usually rely on small particles (Merz 1984). The feeding biology of the crinoid *P. kerguelensis* is not known (but see Liddell 1982, Kitazawa and Oji 2010). Deposit‐feeding invertebrates are generally understood to rely on large phytoplankton and benthic diatoms, and the reduction of *Sterechinus neumayeri*,* Abatus nimrodi*, and *Gersemia antarctica* (reviewed by McClintock 1994, Slattery et al. 1997) in the west sound, and *Odontaster validus* and *Sterechinus neumayeri* in the east sound are also consistent with a shift of productivity from large to very small particles.

The ecological succession at Salmon Bay followed a mass mortality of most benthic animals resulting from a flood depositing over 50 cm of sediment on the bottom. The patterns we observed some 10 years after the flood included species with effective dispersal such as the sponge *Homaxinella balfourensis*, which are known to settle in dense assemblages and grow very fast (Dayton 1989), and the polychaetes *Perkinsiana littoralis* and *Serpula narconensis* that also had strong recruitment at Explorers Cove.

The *Laternula elliptica* recruitment observed at Salmon Bay in 2010 contrasts with the complete elimination of the population in our surveys at Explorers Cove with no sign of *L. elliptica* recruitment there in 2010. We speculate that the increased settlement at Salmon Bay in contrast to Explorers Cove reflects the lack of the various larval filters considered in Dayton et al. (2016b) that may have been reduced by the sedimentation associated with the flood discussed in Dayton et al. (2016a).

*Adamussium colbecki* was extremely abundant in Salmon Bay near the ice wall in 1988, probably with densities >100 individuals/m as reported by Stockton (1983, 1984). They also occurred at high densities at 18 and 21 m in 1988, but they were completely gone in 2010. Similarly, *Sterechinus neumayeri* had a relatively high density in 1988 but was entirely missing in 2010. This parallels their decline in Explorers Cove, but at Salmon Bay probably reflects the sedimentation. *Abatus nimrodi* was relatively common at 18 and 21 m in 1988 and was also common in the deeper depths in 2010. It is interesting to note that both *L. elliptica* and *A. nimrodi* brood their larvae, yet both also have excellent dispersal abilities. The Bryozoa *Camptoplites* sp. and *Hornera* sp. were very rare in 1988 but very common in 2010, clearly reflecting high recruitment and probably a fondness for tiny phytoplankton and bacteria hypothesized to be the dominant components of their food. The carnivores *Ophionotus victoriae*,* Ophiosparta gigas*, and *Parborlasia corrugatus* were very patchy but relatively abundant in 1988, and still patchy and relatively abundant in 2010.

What environmental factors may have driven these changes? The first and most obvious correlation involves the shift in current patterns that resulted from grounded icebergs blocking McMurdo Sound starting in 2000. Arrigo et al. (2002), Thrush and Cummings (2011), Conlan et al. (2010), and Kim et al. (2018) review what is known about the oceanography over this decade, and report mega‐icebergs were grounded beginning in 2000 and 2001. The northern two‐thirds of one broke free in 2003 and the rest broke free in 2006. The mega‐iceberg emplacement in 2000 was followed by several years during which the sea ice did not clear from southern McMurdo Sound. During this period the sea ice thickened from the normal 2.3–5.2 m (Kim et al. 2018), which blocked most light transmission. The thick sea ice and increased snow cover considerably reduced in situ benthic productivity (Dayton et al. 1986, Kim et al. 2018), resulting in a further decrease in food available to the benthos.

The mega‐icebergs were correlated with reduced advection and growth of the large phytoplankters that usually characterize the east side of the sound (Arrigo et al. 2002, 2015, Seibel and Dierssen 2003). We conclude that the grounded icebergs changed the current patterns for at least four years by blocking and reducing the southerly flow from the Ross Sea along the east side of the sound, and by trapping the northerly flowing waters that originate beneath the Ross Ice Shelf and move along the west side of the sound (Barry 1988). Thus, it is likely there was decreased advection of primary productivity from the open Ross Sea into McMurdo Sound in late summer, and decreased food available to the benthos.

The observed community changes suggest not just a response to a generalized decrease in food, but a shift to taxa capable of utilizing smaller particulates. In addition to the results of Seibel and Dierssen (2003), we outline indirect support for a shift in the planktonic community from one dominated by microphytoplankton in the fall, to smaller nano‐ or picoplankton throughout the summer. Over the last 30 yr, there has been a notable decrease in spring/summer water clarity under the fast ice in McMurdo Sound (S. Kim, P. K. Dayton, R. Robbins, *personal observations*). In the 1980s and prior, underwater visibility of over 300 m in the beginning of the austral spring was common. By the 2010s, visibility that exceeded 100 m was uncommon and it was often <60 m (Dayton et al. 2016b). This is likely also associated with changes in the timing of sea ice retreat across the decades with earlier onset of summer conditions more recently. Decreasing water clarity is consistent with enhanced phytoplankton blooms indicated by Arrigo et al. (2015) during the summer.

The springtime waters do not appear to have the green or brown tint that would indicate increased abundance of diatoms or *Phaeocystis* spp., the dominant local phytoplankters that usually bloom late in the summer (Smith and Asper 2001). Instead, the tint is white/blue, suggesting that the particulates are suspended glacial flour (Domack et al. 1994, Capello et al. 2004) and/or nano‐ or picoplankton (Morel 2008). We hypothesize that an increase in iron, released from more rapidly melting glaciers and ice shelves (Hawkings et al. 2014, Arrigo et al. 2015, Dayton et al. 2016a, Gooseff et al. 2017) has contributed to a shift in primary producers. In spring, McMurdo Sound contains a significant fraction of water derived from beneath the Ross and McMurdo ice shelves (Lewis and Perkin 1985) that is associated with enhanced concentrations of iron (sensu Hawkings et al. 2014), a primary limiting nutrient in the Southern Ocean (Martin 1992, Arrigo et al. 2015). Recent increases in melt‐water from the Ross and McMurdo ice shelves (Pritchard et al. 2012, Rignot et al. 2013) and other glaciers (Mikucki et al. 2004, 2015) may be contributing more iron and freshwater to McMurdo Sound in the spring. Nanoplankton may be iron limited, though picoplankton may not be (Wells et al. 1994). In the spring when the fast ice is thick, and the amount of incident light to the water column is below the threshold for microphytoplankton growth, the smaller phytoplankters may still be productive (Saggiomo et al. 2000, Dennett et al. 2001), utilizing iron sourced from glacier and ice shelf melt. Dayton et al. (2016b) explicitly discuss the hypothesis that the regime shift is associated with much smaller phytoplankton. Smith et al. (2014) note that microplankton in Antarctic waters are understudied.

In addition to the nutrient changes, fresh water, sourced from ice shelf and glacial melt, has the potential to alter sea ice duration and distribution (Bintanja et al. 2013). Attendant changes in light availability resulting from increased sea ice cover as well as limiting nutrients can alter the species composition and timing and growth of phytoplankton (Arrigo et al. 2010, 2015). Montes‐Hugo et al. (2009) described regional changes in the Western Antarctic Peninsula and suggested a strong relationship between ice cover and reduced size of the phytoplankton. Thus, in addition to increased iron input, eight years of heavy ice cover could have had a similar effect on the phytoplankton fauna in McMurdo Sound.

The only test of our hypothesized mechanism explaining these changes would be additional research focused on both the relevant oceanography and future changes at our benthic study sites. The authors offer as much help as possible to find the transects reported here in hopes that others will find support to continue and expand these studies.

Ecological resilience involves understanding both the potential perturbations and the recovery processes. Here and in our previous paper (Dayton et al. 2016b), we consider the environmental factors associated with the changes in the benthic fauna of McMurdo Sound. Anthropogenic change is both ongoing and inevitable with many unpredicted consequences. It is important to understand the processes resulting in the changes. Ecological perturbations and consequences can be subtle and may result, for example, from shifts in the size of the food particles and/or shifts in ecological barriers to recruitment that can profoundly impact community recovery following disturbance. Better understanding of ecological resilience relies on increased knowledge of all of these processes.

## Data Availability

Data are available from the EDI Data Portal at https://doi.org/10.6073/pasta/a2291228efb3d6639fb8f55ef96566bc and https://doi.org/10.6073/pasta/053de1fb427e26ce37b617447c5778e8.

## Note

1
http://www.peterbrueggeman.com/nsf/fguide/index.html
