# Supplementary material for: Benthic responses to an Antarctic regime shift: food particle size and recruitment biology
Source: Ecol Appl. 2019 Jan 2;29(1):e01823. doi: 10.1002/eap.1823 (PMC6850755; doi:10.1002/eap.1823)
Supplement: Supplementary file 1 [file EAP-29-na-s001.pdf]

Supporting Information. Benthic responses to an Antarctic regime shift: food particle size and recruitment biology. Paul K. Dayton, Shannon C. Jarrell, Stacy Kim, P. Ed Parnell, Simon F. Thrush, Kamille Hammerstrom, James J. Leichter. *Ecological Applications.* 2018.

Appendix S1

Over the years we have made natural history observations that can fill in details and help understand other patterns and relationships that might be observed in the future. This appendix offers ancillary natural history background to this paper. We hope the information is useful to future ecologists working in this area.

Movement and behavioral observations

A motile hexactinellid sponge?

We established many permanent transects at Cape Armitage in 1974, but the transects were almost completely buried by sponges and sedimentation by 2010. Essentially all of the "volcano" sponges from the 1960s and '70s had died by 2010. A photo of a rare exception was on a transect at about 50 m depth in which three *Rossella racovitzae* photographed in 1975 were still alive in 2010. The interesting enigma is that one of the sponges had moved about 50 cm. *R. racovitzae* has long anchoring spicules (see Dayton et al. 2016b) which makes this observation particularly enigmatic. We have observed an *Anoxycalyx joubini* in the shallow anchor ice zone (25m) at Cape Armitage with anchor ice that had lifted the sponge slightly off the bottom. It was moving a little and presumably with enough anchor ice it could have moved a significant distance. Indeed, recent photographs of the large *Anoxycalyx joubini* on the gangplank at 23 m depth show several of the sponges completely missing, presumably carried away by anchor ice. However, the *R. racovitzae* in Appendix S1 Figure S1 is at 50 m, much below the normal depth of anchor ice.

The "volcano sponge" complex represents the volcano shaped, usually white, hexactinellids that were lumped in the 1960s (see supplement B in Dayton et al. 2016b). This paper reports surprisingly fast growth rates for *Anoxycalyx joubini* and a single *Rossella fibulata*, but the other "volcano" sponges did not recruit onto the artificial structures and we have no information about their growth rates. Appendix S1 Figure S1, showing rather slow growth over 35 years, suggests that *R. racovitzae* has a much slower growth rate than some of the other hexactinellids discussed in Dayton et al. (2016b).

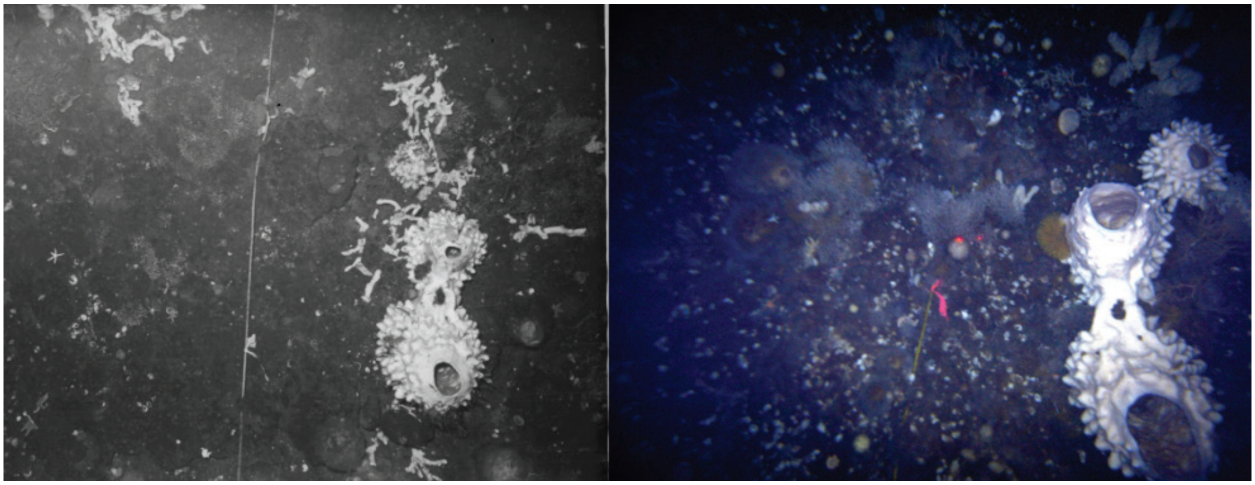

Appendix S1 Figure S1. Three *Rossella racovitzae* photographed in 1975 and 2010 at 50 m depth at Cape Armitage.

Rates of movement of actively foraging predators at Explorers Cove

Predator diets have been discussed in previous papers. Here we add relevant behavioral observations. In 1977 we made an effort to evaluate the movement of the brittle star, *Ophionotus victoriae,* that seemed to be very active. And, in fact it was, and so too were *Sterechinus neumayeri*.

Movement of a few active species was studied using photographic surveys separated by two days at Explorers Cove. The movement of individual animals identified in successive photos was evaluated as a straight-line distance over the two days. While the species could be identified, it was sometimes not possible to identify a specific individual, but individual *O. victoriae* and S*. neumayeri* could usually be identified by color or epifauna on their shells or spines. There were many situations in which an individual moved out of the photo entirely and in those cases the minimal distance to the edge of the photo was used. For this reason, the estimates of movement distances are very conservative.

Ophionotus victoriae: Of 106 individuals, 80% moved enough to measure their movement. Of those movements that could be tracked, 54 individuals moved a total of 2,385 cm in two days with an average daily movement of 22.1 cm per day. The conservative measure of those 31 that moved out of the picture for a total of 1053 cm was 17.0 day^{-1}. Conservatively, those that were moving covered a minimum of about 20 cm day^{-1}.

Sterechinus neumayeri: The common sea urchin turned out to be much more motile than we anticipated with a full 86% of the 36 individuals observed moving considerable distances. Those that covered distances we could measure moved 30.5

cm day^{-1} and those that went off the photos covered a minimum of 28.2 cm day^{-1}. A conservative average daily movement rate for *Sterechinus neumayeri* was almost 30 cm day^{-1}.

Abatus nimrodi: Only six heart urchins were observed in this study; two were not moving and the other four moved a total of 103 cm in two days or about 13 cm day^{-1}.

Adamussium colbecki*:* Only 17% of the 106 scallops moved over the two days. They all moved out of the picture with a minimal distance of 20 cm day^{-1}. Once the scallops launch themselves, they continue to flap for some time, once coming a full 10 - 15 m off the bottom, thus they probably move a considerable distance. It appeared that each of the 18 scallops that moved responded to an *O. victoriae* or *S. neumayeri* coming in contact.

Feeding observations

Isotealia antarcticus is a common anemone along the east side of McMurdo Sound where the primary food appeared to be various jelly fish that ran aground (Dayton et al. 1970). In 1975 we observed a single large *I. antarcticus* on a rock at 30 m in Explorers Cove. The large anemone was accompanied by an asteroid, *Notasterias armata* eating an *Adamussium colbecki*. There was a trail of dead *A. colbecki* shells in front of the anemone suggesting that it had been feeding on scallops that had probably escaped from various predators in the vicinity by swimming up off the bottom but had landed on the anemone, eventually named Miss Piggy. The anemone was observed on the same rock through the 1970s and 1980, always accompanied by a *N. armata.*

While rare, there is a risk to the scallop escape behavior because they may become food for any anemones in the vicinity. Appendix S1 Figure S2 shows Miss Piggy in 2010, still sitting on the same rock after 35 years. The photograph also shows her accompanied by a *N. armata*. The slow sedimentation has covered many shells, and we estimate the anemone has been eating scallops escaping *O. victoriae* for much longer. A photograph from a different angle in 1985 shows Miss Piggy to be as large as she is in this figure and we believe she was full grown in 1975. We don't know if it is the same starfish, but it is rare in this habitat and we presume that it sticks around to take advantage of Miss Piggy's sloppy feeding.

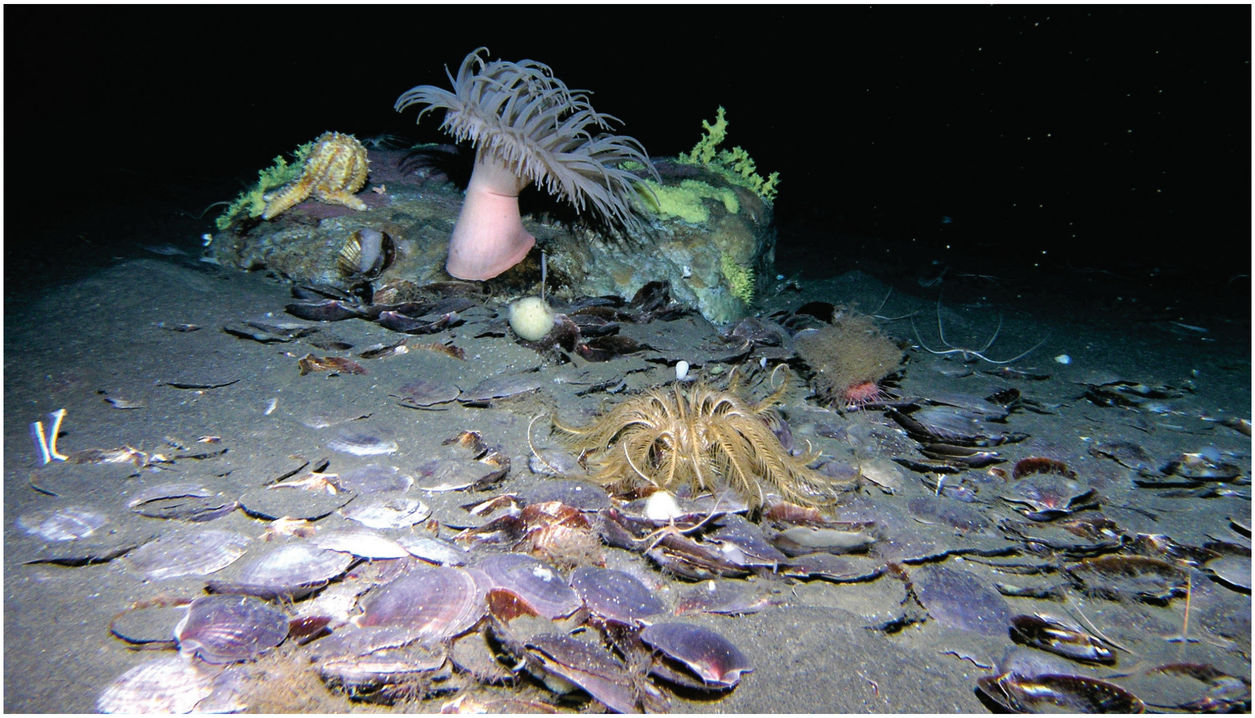

Appendix S1 Figure S2. Miss Piggy, *Isotealia antarctica*, observed feeding on scallops between 1975 and 2010.

The large nermertean worm *Parborlasia corrugatus* appears inactive, but when exposed to a chemical stimulus it becomes extremely active as seen in the time-lapse photography here:

https://www.youtube.com/watch?v=HG17TsgV_qI

This classic BBC production was done at Little Razorback Island, McMurdo Sound (Norbert Wu, personal communication) and represents a highly unusual situation with extremely high densities of asteroids, echinoids and very active nemerteans responding to the stimulus of dead Weddell seal pups. But it does dramatically document the highly active foraging of *Parborlasia corrugatus.* The diet of this nemertean is well described, to which we add our observation of attack on spawning *Laternula elliptica* in which the clams release sperm that flows along the sea floor where it can be available to female *L. elliptica.* The *P. corrugatus* in the area spring to life and very quickly follow the sperm trail to the clam and go down the hole to eat the clam.

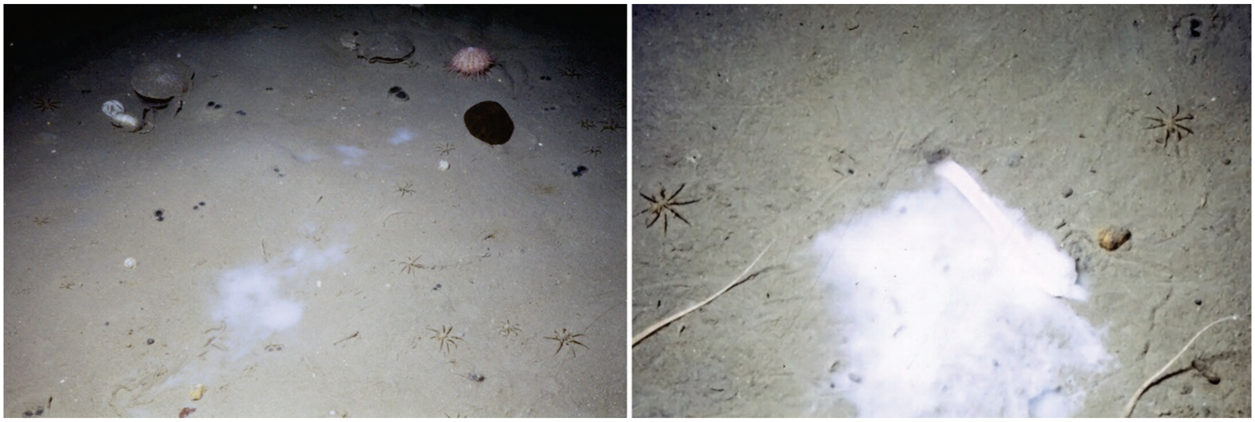

Appendix S1 Figure S3. A *Laternula elliptica* releasing sperm at 30 m depth at Salmon Bay in 1989 (left) and the nemertean, *Parborlasia corrugatus,* moving down into the clam burrow (right).

Interesting invertebrate observations

John Dearborn's 1965 PhD thesis represents an extremely extensive biological collection, and is still the definitive word on the species richness of McMurdo Sound. Here we discuss a few interesting additions that may be useful to future workers.

Several large full grown *Anthomastus* sp were observed in Salmon Bay in 2010. Assuming the 50 cm of sediment from the flood was about 2001, this demonstrates a remarkably fast growth rate for a typically deep-sea alcyoniid Cnidaria.

Distaplia cylindrica is a widespread Antarctic colonial ascidian with stalks approaching 10 m in length. Before 2010 we only saw one stalk that had broken free and drifted against the shore at Cape Armitage in 1967. It was considered a deep-water species at the time, but in 2010 several were seen both at Cape Armitage and Explorers Cove (Appendix S1 Figure S4). Some at Cape Armitage were large (< 7 m in length), often with very small ones in the vicinity reflecting settlement of the non-feeding larvae. Interestingly, we saw no large individuals at Explorers Cove but there were scattered individuals in the 10-30 cm range implying that the larvae did disperse into this area after 1989.

A stalked solitary tunicate (Appendix S1 Figure S5) is another interesting ascidian that settled after the flood in Salmon Bay (Dayton et al. 2016a). It has been tentatively identified as *Pyura bouvetensis* by Craig Smith. This tunicate is extremely rare in McMurdo Sound and it appeared that there were several other small individuals in the area where this photo was taken. Like *Distaplia*, this ascidian appears to have recruited into the area rather recently as it was not seen in any of our study sites before 2010.

The lollipop sponge, *Stylocordyla chupachups*, is another deep-water species that showed up in our study areas for the first time after the Salmon Bay flood. We observed 3 very small sponges that we believe were *S. chupachups*.

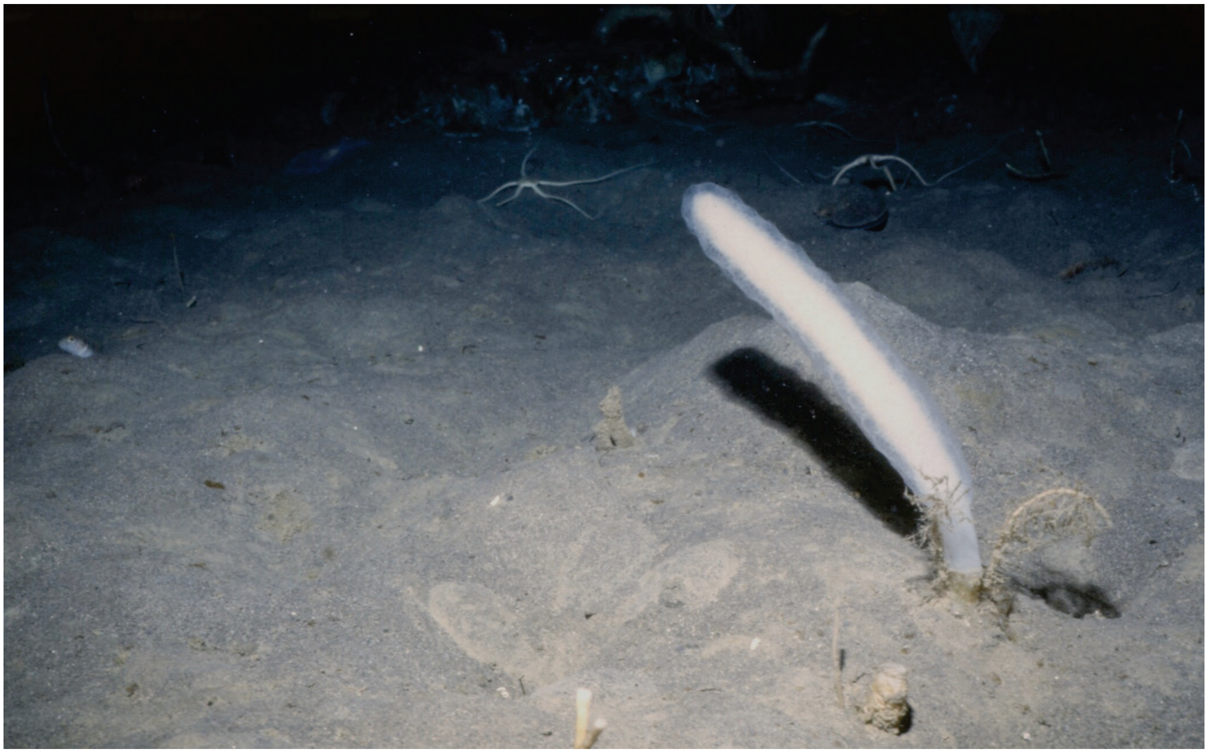

Appendix S1 Figure S4. Young *Distaplia cylindrica* at 35 m depth at Explorers Cove. Note mounds that appear to be made by Thalassinidae shrimp in the background.

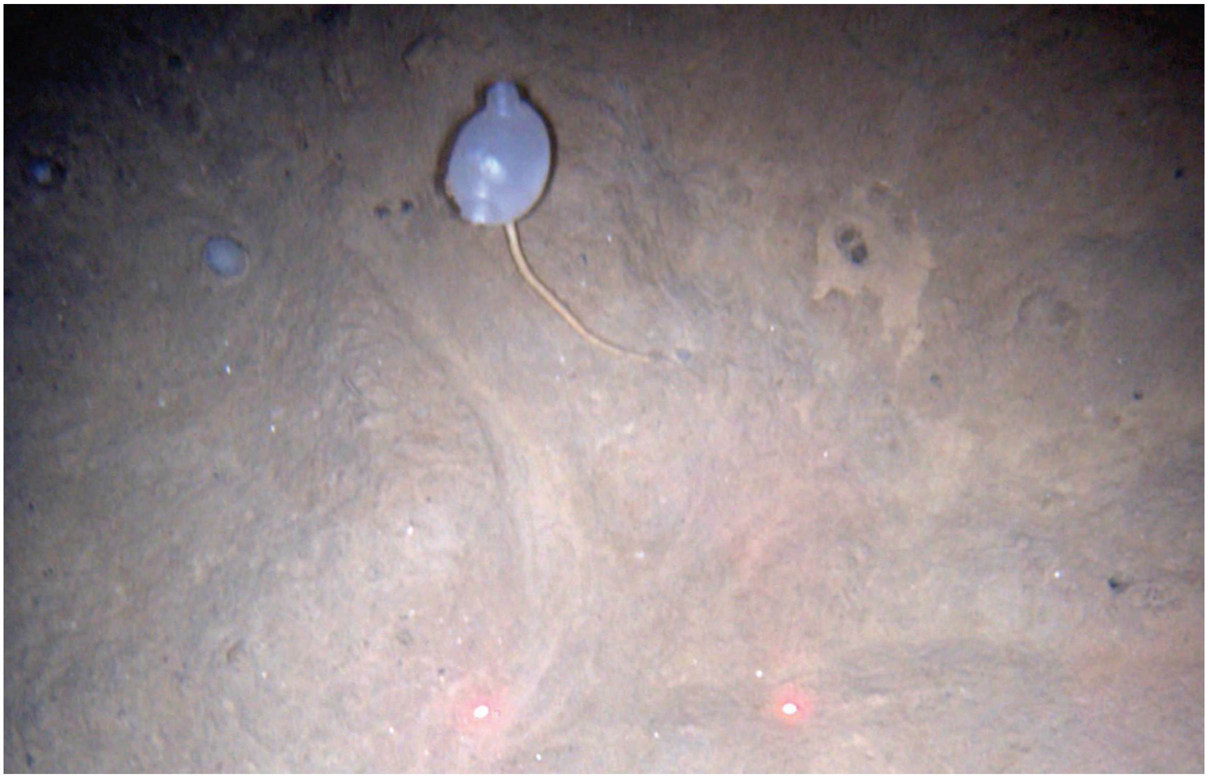

Appendix S1 Figure S5. A stalked tunicate observed for the first time at Salmon Bay.

Malacobelemnon daytoni is a small white pennatulacean octocoral that has been very abundant in Potter Cove in the South Shetland Islands (Servetto, et al. 2013). Two photos appear to include examples this small soft coral.

We had not expected to find Thalassinidae shrimp in the high Antarctic, but in fact mounds that appear to be made by these shrimp were common in patches in both Explorers Cove and Salmon Bay (Appendix S1 Figure S4). We knocked the top of one of the mounts and sand was blown out in the same way that Thalassinidae shrimp do elsewhere. Unfortunately it was not possible to collect the deep burrowing shrimp.

Additional Observations of Interest

Finally, three additional natural history observations have been published or are in press in Antarctic Science. These include an unexpected photograph of what seems to be a hagfish at Salmon Bay (Dayton, 2018), long-term persistence of wood on the bottom (Dayton, et al., 2018) and bacterial resistance to scavengers (Dayton et al., in press).

Literature Cited

Dayton, P.K. and K. Hammerstrom. 2018. A hagfish at Salmon Bay, McMurdo Sound, Antarctica? Antarctic Science 30 (4): 243-244. doi:10.1017/S0954102018000202

Dayton, P. K. G. A. Robilliard, J. S. Oliver, S. Kim, K. Hammerstrom, K. O'Connor, J. Fisher, J. Barber, and S. Jarrell. 2018. Long-term persistence of wood on the sea floor at McMurdo Sound, Antarctica. Antarctic Science, 1-2. doi:10.1017/S0954102018000305

Dayton, P.K. , J. S. Oliver, S. F. Thrush, K. Hammerstrom. In press. Bacteria defend carrion from scavengers. Antarctic Science.

Dayton, P. K., G. A. Robilliard, and R. T. Paine. 1970. Benthic faunal zonation as a result of anchor ice at McMurdo Sound, Antarctica. Pages 244-258 *in* M. Holdgate, editor. Antarctic Ecology. Academic Press, London, UK.

Dayton, P. K., G. A. Robilliard, R. T. Paine, and L. B. Dayton. 1974. Biological accommodation in the benthic community at McMurdo Sound, Antarctica. Ecological Monographs 44:105–128.

Dayton, P. K., K. Hammerstrom, S. C. Jarrell, S. Kim, W. Nordhausen, D. J. Osborne, and S. F. Thrush. 2016a. Unusual coastal flood impacts in Salmon Valley, McMurdo Sound, Antarctica. Antarctic Science 28(4):269-275.

http://dx.doi.org/10.1017/S0954102016000171

Dayton, P., S. Jarrell, S. Kim, S. Thrush, K. Hammerstrom, M. Slattery, and E. Parnell. 2016b. Surprising episodic recruitment and growth of Antarctic sponges: implications for ecological resilience. Journal of Experimental Marine Biology and Ecology 482:38–55.

Dearborn, J.H. 1965. Ecological and faunistic investigations of the marine benthos at McMurdo Sound, Antarctica. Doctoral dissertation, Stanford University, California.

Servetto. N., L. Torre, and R. Sahade. 2013. Reproductive biology of the Antarctic "sea pen" *Malacobelemnon daytoni* (Octocorallia, Pennatulacea, Kophobelemnidae). Polar Research 32, 20040, http://dx.doi.org/10.3402/polar.v32i0.20040
